# Effect of calorie restriction and intermittent fasting on glucose homeostasis, lipid profile, inflammatory, and hormonal markers in patients with polycystic ovary syndrome: a systematic review

**DOI:** 10.3389/fnut.2024.1362226

**Published:** 2024-04-05

**Authors:** Anam S. Kalsekar, Dana N. Abdelrahim, MoezAlIslam E. Faris

**Affiliations:** ^1^Department of Clinical Nutrition and Dietetics, College of Health Sciences, University of Sharjah, Sharjah, United Arab Emirates; ^2^Health Promotion Research Group, Research Institute of Medical and Health Sciences (RIMHS), University of Sharjah, Sharjah, United Arab Emirates; ^3^Nutrition and Food Research Group, Research Institute of Medical and Health Sciences (RIMHS), University of Sharjah, Sharjah, United Arab Emirates; ^4^Healthy Aging, Longevity and Sustainability Research Group, Research Institute of Medical and Health Sciences (RIMHS), University of Sharjah, Sharjah, United Arab Emirates

**Keywords:** time-restricted eating, alternate-day fasting, Ramadan fasting, diurnal fasting, gynecology, infertility

## Abstract

**Background and objective:**

Polycystic ovary syndrome (PCOS) is a complex hormonal disorder that leads to ovarian cysts, irregular ovulation, and hormonal swings in women. It is a complex and heterogeneous condition that affects 4 to 20% of women of reproductive age worldwide and relates to reproductive, metabolic, and psychosocial dysfunction. Dietary and lifestyle modifications have been proposed to play a central role in the management of PCOS. This study aimed to provide a comprehensive systemic overview of the existing literature on the effects of intermittent fasting (IF) and calorie restriction (CR) regimens on disease markers of PCOS.

**Designs and methods:**

Several databases, such as CINAHL, Cochrane, EBSCOhost, EMBASE, Google Scholar, ProQuest Medical, PubMed/MEDLINE, ScienceDirect, Scopus, and Web of Science databases were searched for clinical trials and observational studies examined the effects of IF regimens such as time-restricted eating and Ramadan model of IF (RIF) on glucose homeostasis, lipid profile, inflammatory and hormonal markers in patients with PCOS.

**Results:**

This systematic review solicited three articles, comprising a collective sample size of 75 females diagnosed with PCOS. The studies were published between 2015 to 2023 and were undertaken in three countries: China, Turkey, and Iran. The research articles examined the effects of intervention with IF and CR on PCOS-related parameters such as anthropometric measures and biochemical tests which included enzymes, glycemic control, lipid profile, hormonal, and oxidative stress, and inflammatory markers. The articles yielded mixed results, with two of them showing significant changes across all tested parameters. One of the three studies did not exhibit any significant changes.

**Conclusion:**

Very limited studies examined the relationship between IR and CR with markers of PCOS. Further well-controlled studies need to be undertaken the combined results from the limited studies illustrate the intricate and diverse nature of IF, including the RIF, and its influence on measurements of body composition and biochemical markers related to PCOS.

## Introduction

Polycystic ovary syndrome (PCOS) is a prevalent endocrine condition distinguished by persistent anovulation, biochemical and/or clinical hyperandrogenism, and the presence of polycystic ovary morphology (1). The World Health Organization (WHO) estimates that PCOS affects more than 116 million women worldwide (1). This disease has significant clinical implications and can lead to health issues related to the accumulation of adipose tissue, including obesity, insulin resistance (IR), metabolic syndrome (MetS), and type 2 diabetes mellitus (T2DM) (2). Insulin resistance can be elucidated by the necessity of elevated insulin levels to support metabolic processes, as well as its involvement in mitogenic and reproductive functions (3). Previous studies have indicated that a substantial proportion of women with PCOS experience impaired glucose tolerance and IR and are at increased risk for developing T2DM (3). In general, the presence of abdominal obesity or visceral adiposity in individuals with PCOS may contribute to IR, potentially triggered by subclinical, systemic low-grade inflammation. Recent research has determined that obesity is the primary risk factor for IR in persons diagnosed with PCOS (3). Another significant issue related to PCOS is hyperandrogenism, a condition that is also associated with IR. The premature secretions of androgen during early stages are commonly regarded as a characteristic feature of PCOS and are believed to contribute to the development of IR in preceding stages (3).

Sarahian et al. (4) propose that PCOS can arise from a combination of lifestyle, genetic, and prenatal influences, hence giving rise to a range of effects with differing magnitudes. The risk of PCOS is heightened by an unhealthy lifestyle and dietary choices, or exposure to infectious agents (5, 6). Environmental factors, such as physical exercise, dietary and lifestyle behaviors, and food choices exhibit significant variability among different populations.

Frequently, PCOS treatment focuses on the management of underlying symptoms, typically involving progestin therapy and a combination of birth control medications (7). Furthermore, the implementation of lifestyle modifications, specifically the adoption of healthful dietary practices and nutritious food choices, is highlighted as a viable approach for effectively managing the condition (5). The overall condition is improved by the combination of pharmacological treatments and dietary and lifestyle modifications. According to Xu and Qiao (3), lifestyle therapies, including exercise, weight loss, and nutrition therapy, have demonstrated favorable results in individuals with PCOS.

The implementation of nutrition therapy for weight reduction in women diagnosed with PCOS has been found to have a substantial influence on metabolic conditions. Previous research has demonstrated that patients with obesity who lose between 5 and 10 percent of their body weight (BW) experience significant health benefits (8). In recent decades, fasting regimens, especially what is commonly known as intermittent fasting (IF), have emerged as a non-pharmaceutical lifestyle approach in integrative medicine and a means to reduce and control weight to enhance health (9). The implementation of IF has been shown to have the potential to reduce adiposity and improve IR through decreased calorie intake and metabolic reprogramming. It can result in several positive health outcomes, such as enhanced metabolic efficiency, enhanced cognitive acuity, and an extended life span (10, 11).

Ramadan intermittent fasting (RIF), is another type of IF regimen. During Ramadan, adult Muslims are mandated to refrain from food and drink for 12–22 h during the day, depending on the season and geographical location. This fasting pattern is followed consistently for 29–30 days. Multiple studies, including original research, systematic reviews, and meta-analyses, have provided evidence that RIF is linked to decreased BW, body fat mass especially visceral fat, serum lipids, and other cardiometabolic risk factors, inflammatory and oxidative stress markers, with slight improvements in glucometabolic regulation and liver function tests (12–19). Most, if not all, of these aforementioned factors, improved upon RIF have been implicated in the etiopathogenesis of PCOS in variable degrees and dimensions (20–22).

Based on the above literature, and previous research examining the impact of different models of IF on various metabolic, hormonal, and inflammatory markers and health indicators, as well as their reported protective effect, it is hypothesized that CR and IF will improve metabolic parameters in PCOS patients. Therefore, this review aims to provide a comprehensive summary of the existing literature on the effects of Ramadan and non-Ramadan IF and CR regimens on PCOS markers.

## Methods

This systematic review followed Preferred Reporting Items for Systematic Reviews and Meta-Analyses (PRISMA) to guide the reporting of the findings (23).

### Review question

The PICO model was employed to expand the return from the review and applicability of the data collected (Table 1).

**Table 1:** PICO Model.

Population	Females above the age of 18 years diagnosed with PCOS using Rotterdam criteria
Intervention	Any form of intermittent fasting regimen such as time-restricted eating (TRE), alone or associated with pharmacological therapy, exercise, and weight loss. To include 16:8 method, 18:6 method, 5:2 diet, alternate day fasting, intermittent fasting, Ramadan model of intermittent fasting, or other suitable religious intermittent fasting regimens
Comparator	Comparison to the usual *ad libitum* diet or no dietary or non-fasting intervention, standard treatment-as-usual, and pharmacological therapy
Outcomes	Any PCOS metabolic, hormonal, inflammatory, or anthropometric markers including insulin levels, C-peptide concentrations, glucagon, insulin-like growth factor 1 (IGF-1), glycated hemoglobin (HbA1c), homeostatic model assessment for insulin resistance (HOMA-IR), fasting blood glucose, oral glucose tolerance test (OGTT), lipid profile such as and serum lipid profile parameters, low-density lipoprotein (LDL), high-density lipoprotein (HDL).; hormonal markers such as sex hormone binding globulin (SHBG), follicular stimulating hormone (FSH), luteinizing hormone (LH)

### Database searches

An electronic search of the databases was conducted of the Google Scholar, PubMed/MEDLINE, EBSCO-host, CINAHL, ScienceDirect, Cochrane, ProQuest Medical, Web of Science, and Scopus databases for targeted studies published from 1970 to the end of November 2023. The keywords were taken from a bibliometric analysis paper by Obaideen et al. (24). Accordingly, the following search criteria were applied: TITLE-ABS-KEY (“intermittent fasting”) OR (“Ramadan”) OR (Ramadhan) OR (Ramazan) OR (“Islamic fasting”) OR (“diurnal fasting”) OR (“Ramadan intermittent fasting”) OR (“Ramadan diurnal intermittent fasting”) OR (“consecutive 30 days of fasting”) OR (“religious fasting”) AND (“PCOS” OR “polycystic ovary syndrome” or “polycystic ovarian syndrome”).

### Inclusion criteria

Both observational and experimental studies investigating the impact of CR, IF including RIF on markers related to PCOS were included. The inclusion criteria for research articles were as follows: (1) experimental and observational studies; (2) adult female participants (>18 years) diagnosed with PCOS; (3) endpoints that included changes in at least one PCOS diagnostic biomarker before and after following the CR or IF regimen.

### Exclusion criteria

**Figure 1:**
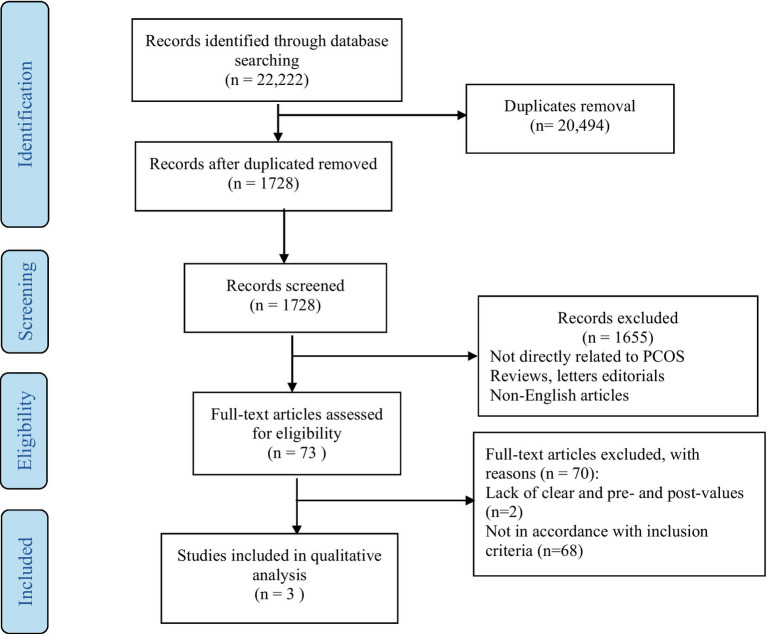
PRISMA flowchart for systematic review search.

Specific exclusion criteria were applied to eliminate any potential quality or methodological issues: (1) studies involving patients with any other disease apart from PCOS; (2) lacking full-text; (3) non-English language; (4) lack of clear and pre and post data; (5) editorials, abstracts case reports, and review articles; and (6) non-peer-reviewed and unpublished data. The steps for study selection are summarized in the PRISMA flow diagram (Figure 1).

### Main outcomes and measures

The main outcome was to report the effect of practicing CR and IF regimens including RIF-induced changes in PCOS-related parameters, namely anthropometric, glycemic control, lipid profile, hormonal, oxidative, and inflammatory parameters. The first step of screening was examining all titles and abstracts to exclude irrelevant publications.

Two authors (AK and DA) independently screened the titles and abstracts of identified studies and assessed them for eligibility against the inclusion criteria. Discrepancies during screening were resolved by the principal investigator (MF). All titles and abstracts were screened to exclude irrelevant publications. To standardize data collection, the researchers reported and tabulated the study characteristics, including the main author’s name, study country, year of publication, sample size, type of fasting, study design, and main findings for the examined outcomes.

## Results

Three studies with a total of 75 participants were included in this systematic review. Details of the study authors, year of publication, country, design and type of IF regimen, sample size, age, and biomarkers tested, and the effects of CR and IF regimens are shown in Table 2. The included studies were conducted in 3 different countries, i.e., China, Turkey, and Iran. The parameters included in these studies were as follows: anthropometrics and biochemical tests including enzymes, glycemic control, lipid profile, hormonal parameters, oxidative stress, and inflammatory markers.

**Table 2:** Characteristics and major findings of the included studies on the effect of intermittent fasting on patients with PCOS.

Author, publication year	Country	Sample Size (*n*)	Age range (average)	Type of fasting	Study Design	PCOS Markers						
						Anthropometric measurements	Glycemic control	Lipid Profile	Hormones	Liver enzymes	Oxidative stress and inflammatory markers	Major findings
Li et al. (25)	China	18	18–31	IF	Clinical trial	BW (kg) BMI (kg/m^2^) WHR SMM (kg) BFM (kg) BF% VFA (cm^2^)	FG (mmol/L) FINS (μU/mL) AUCIns (mU/L*min) AUCGlu (mmol/ L*min) AUCIns/AUCGlu HOMA-IR IGF-1 (ng/mL)	TG (mmol/L) TC (mmol/L) LDL-C (mmol/L)	TT (ng/mL) SHBG (nmol/L) FAI (%) LH (mIU/mL) FSH (mIU/mL) LH/FSH	AST (U/L) ALT (U/L)	hs-CRP (mg/L)	BW ↓, BMI ↓, BFM ↓, BF% ↓ VFA ↓ ALT ↓ FINS ↓, AUCIns ↓ AUCIns/AUCGlu ↓, HOMA-IR ↓ IGF-1 ↑, TT ↓, SHBG ↑, FAI ↓ CRP ↓
Feyzioglu et al. (26)	Turkey	30	21–33	IF	Clinical trial	BMI (kg/m^2^) WHR	FINS (μU/mL) FG (mg/dL) HOMA-IR IR (>2.4) HbA1c	HDL-C (mg/dL) LDL-C (mg/dL) TG (mg/dL)	FSH (mlU/mL) LH (mlU/mL) E2 (mlU/mL) TSH mlU/mL Prolactin (ng/mL) TT (ng/dL) Free testosterone (pg/mL) SHBG (nmol/L) FAI (%) Hyperandrogenism (≥8) DHEAS (μg/dL) AMH (ng/mL)		Calprotectin (μg/g)	BMI ↓, WHR↓ FBG ↓, FINS ↓, HOMA-IR ↓, IR ↓ HbA1c ↓ TG ↓, LDL ↓, HDL ↑, TT ↓ SHBG ↑, FAI ↓, LH ↓, FSH ↓, E2 ↓ TSH ↑, Prolactin ↓, Free Testosterone ↓, DHEAS ↓, AMH ↓, Hyperandrogenism ↓ Calprotectin ↓
Asemi et al. (27)	Iran	27	18–40	RIF	Cross-section study	Height (cm) Weight (kg) BMI (kg/m^2^)	FG (mg/dL) FINS (IU/mL) HOMA-IR HOMAB QUICKI	TG (mg/dL) VLDL-C (mg/dL) TC (mg/dL) LDL-C (mg/dL) HDL-C (mg/dL)			NO (mol/L) TAC (mmol/L) GSH (mmol/L) MDA (mmol/L) hs-CRP (ng/mL)	NO ↑, GSH ↑

↓ Significantly decreased; ↑ Significantly increased. AMH, anti-mullerian hormone; AUC GIu., area under the curve (AUC) for glucose; AUC Ins., area under the curve (AUC) for insulin; BF%, body fat percent; BFM, body fat mass; BMI, body-mass index; BW, body weight; DHEAS, dehydroepiandrosterone sulfate; E2, estradiol; FAI, free androgen index; FG, fasting glucose; FINS, fasting insulin; FSH, follicle stimulating hormone; GSH, glutathione; HbA1c, glycated hemoglobin A1c; HDL-C, high-density lipoprotein-cholesterol; HOMA-IR, homeostasis model assessment-insulin resistance; hs-CRP, high-sensitivity C-reactive protein; IR, insulin resistance; LDL-C, low-density lipoprotein-cholesterol; LH, luteinizing hormone; MDA, malondialdehyde; NO, nitric oxide; SHBG, sex hormone binding globulin; SMM, skeletal muscle mass; TAC, total antioxidant capacity; TC, total cholesterol; TG, triglycerides; TSH, thyroid stimulating hormone; TT, total testosterone; VFA, visceral fat adiposity; VLDL-C, very low-density lipoprotein-cholesterol; WHR, waist-hip ratio.

### Anthropometric measures

In terms of the results obtained for the anthropometric parameters, Li et al. (25) in their study, reported a significant decrease in all anthropometric parameters, i.e., BMI, BW, BFM, BF%, and VFA (*p* ≤ 0.001), with the exception of WHR and SMM, which showed no significant association with IF. Feyzioglu et al. (26) reported significant reductions in anthropometric parameters, namely BMI and WHR. Asemi et al. (27) did not find any significant effect of RIF on anthropometric parameters.

### Liver enzymes and glucometabolic markers

In terms of enzymes, Li et al. (25) reported no significant effect of IF on enzymes such as UA and AST, while there was a significant decrease in ALT (*p* = 0.027). In relation to the glycemic control parameters, Li et al. (25) reported significant decreases in all glycemic control parameters, i.e., FINS, HOMA-IR, and HbA1c, with the exception of FBG. Similarly, Feyzioglu et al. (26) reported significant decreases in all glycemic control parameters, i.e., FINS, FBG, HOMA-IR, and HbA1c. However, Asemi et al. (27) did not find any significant effect of RIF on glycemic control parameters.

### Lipid markers

Additionally, all the 3 included studies reported on the effect of IF and RIF on the lipid profile. In their study, Li et al. (25) did not find any significant effect of IF on the serum lipids. Similarly, Asemi et al. (27), in their study, did not find any significant effect of RIF on the lipid profile parameters. Conversely, Feyzioglu et al. (26) reported a positive effect of IF on serum lipids, with significant decreases in LDL and TG and a significant increase in HDL.

### Hormonal markers

Li et al. (25) reported no significant effects of IF on hormonal parameters such as LH and FSH, while a significant increase in IGF-1 and SHBG was observed after IF. Alternatively, they found significant reductions in TT and FAI. Feyzioglu et al. (26) reported significant effects of IF on all hormonal parameters, with decreases reported in FSH, LH, E2, Prolactin, TT, FT, FAI, DHEAS, and AMH, while reported significant increases in TSH and SHBG. The study by Asemi et al. (27) did not report any hormonal markers in their study.

### Oxidative stress and inflammatory markers

Li et al. (25) found a significant decrease in inflammatory markers, *hs*-CRP. Feyzioglu et al. (26) found a significant decrease in the oxidative stress marker, calprotectin. Asemi et al. (27) found no significant effect of RIF on inflammatory and oxidative stress markers such as TAC, MDA, and *hs*-CRP. On the other hand, the authors reported significant increases in NO and GSH.

## Discussion

The current study tried to systematically elaborate on the effect of following CR and IF regimens on the disease parameters of PCOS. Only three studies were selected after applying the inclusion/exclusion criteria, a matter that denotes the relatively emerging topic we are currently addressing and the mass need for further studies to be conducted in this regard. The scarcity of studies reviewed is consistent with the finding of Floyd et al. (28), who examined the effect of practicing TRE on insulin levels and insulin sensitivity in patients diagnosed with PCOS. After screening 2,662 studies and assessing 37 eligible studies, only one study was found by Floyd and colleagues.

### Anthropometric markers

Across different studies, the effect of IF on anthropometric measurements has been considered. For instance, Li et al. (25) and Feyzioglu et al. (26) reported remarkable decreases in various anthropometric markers. Whilst, the study by Asemi et al. (27) which studied RIF in particular, revealed no significant relationship between RIF and these anthropometric markers. However, previous studies by Faris et al. (16) and Hooshiar et al. (29) state that RIF lowers visceral adiposity hence it can reduce BW and other parameters in individuals with PCOS. Additionally, Madkour et al. (30), in their study found that RIF resulted in weight loss, and reduced BMI, BF%, and waist circumference. In their study, the beneficial effects of RIF were observed across all subgroups in the study, regardless of age, sex, and fasting duration. Another meta-analysis was conducted by Jahrami et al. (17) to investigate the impact of RIF on BW. It was found that RIF can cause variable changes in BW, body composition, and fat mass. The reduction in BW and visceral adiposity could be attributed to the metabolic shift to ketogenesis and fatty acid oxidation during fasting (15). However, weight changes induced by RIF were found to be mostly reversed post-Ramadan, indicating that weight loss during this period is transient. This difference in findings represents a split landscape; some studies show that IF positively influences body composition, while others fail to back it up.

### Glucometabolic markers and liver enzymes

In terms of enzymes, the included studies reported minimal effect on the liver enzymes. Regarding the glycemic control parameters, two of the included reported a significant reduction in all glycemic control parameters (23, 26). However, Asemi et al. (27) did not find any significant effect of RIF on the glycemic control parameters. Previous studies have also reported such contradictory findings. A systematic review and meta-analysis by Faris et al. (14) examined the effects of RIF on glucometabolic markers in healthy people. The study found that RIF had a minimal impact on these markers and highlighted the influence of various factors such as sex, age, fasting duration, and country on glucometabolic changes. Conversely, Faris et al. (14) reported studies conducted on rodents, which showed that IF improved insulin sensitivity and glucose tolerance, and preserved β-cell mass in obesity-induced diabetes. Likewise, in a study by Carter et al. (31), the researchers conducted a large trial to evaluate the impact of IF versus a continuous energy-restricted diet on glycemic control in individuals with type T2DM. After a year of intervention, both groups showed comparable decreases in HbA1c levels. However, in a study by Faris et al. (16) despite a significant drop in plasma adiponectin levels, no significant change was found in insulin sensitivity or glucose homeostasis markers. Additionally, the study also found a significant increase in apelin levels, which could be responsible for the lack of significant change in insulin resistance, despite an increase in total sugar intake during Ramadan. The study also noted that the level of IGF-1 significantly decreased by the end of RIF, which may be another reason for the non-significant changes in markers of glucose homeostasis.

### Lipid markers

Regarding the lipid markers, 2 of the included studies did not find any significant effect of IF on the lipid profile parameters (23, 27) Conversely, Feyzioglu et al. (26) reported a positive effect of IF on serum lipids, with a significant reduction in LDL and TG and an increase in HDL. Similar findings have been reported by Jahrami et al. (18) in their meta-analysis, they reported that RIF improved lipid profile and coagulation parameters, and these improvements persisted for 4 weeks after fasting. The effects of RIF on the lipid markers are consistent with the impacts of other forms of IF and energy-restricted diets. Additionally, the study also found that IF has cardioprotective effects, possibly due to increased cellular stress resistance, reduced oxidative damage, and changes in the brain-derived neurotrophic factor signaling in the brain.

### Hormonal markers

In terms of the hormonal marker, following IF, Li et al. (25) observed no significant effects of IF on hormonal parameters such as LH and FSH, while significant increases in IGF-1 and SHBG. Alternatively, they found significant reductions in TT and FAI. Likewise, Feyzioglu et al. (26) reported significant effects of IF on all hormonal parameters, with decreases reported in FSH, LH, E2, Prolactin, TT, FT, FAI, DHEAS, and AMH, while reported significant increases in TSH and SHBG. Similar findings have been reported by Cienfuegos et al. (32) in their study which suggested that IF can potentially lower androgen levels, specifically TT and FAI, and increase SHBG in obese premenopausal women, offering a potential treatment for hyperandrogenic conditions like PCOS. Additionally, Han et al. (33) in their study investigated the effects of time-restricted feeding (TRF) on a mouse model of PCOS. The study observed that TRF treatment significantly lowered plasma androgen levels and the LH/FSH ratio in PCOS mice, consistent with other dietary interventions. This suggests that TRF may regulate gonadotropin-releasing hormone secretion, influencing the synthesis of steroid hormones. During fasting, certain gut microbes can use host substrates to produce beneficial metabolites like butyrate, acetate, and mucin stimulants. This suggests that IF’s influence on reproductive hormones may be mediated by alterations in the gut microbiome (32).

### Oxidative stress and inflammatory markers

Among the three included studies, following IF, Li et al. (25) and Feyzioglu et al. (26) found a significant decrease in the inflammatory and oxidative stress markers, *hs*-CRP and calprotectin, respectively. Similar findings have been reported in previous studies. For instance, Faris et al. (16) in their review, reported the effect of RIF on proinflammatory cytokines and oxidative stress markers in both obese and non-obese individuals. The studies included in the review reported a slight decrease in these markers after Ramadan, suggesting short-term protection against low-grade systemic inflammation and oxidative stress. This reduction could be due to weight loss during Ramadan or the lowering of the IGF-1 which is associated with inflammation and oxidative stress. Further, RIF has been linked with reduced serum glucose, insulin, and IR levels in obese individuals with metabolic syndrome. Additionally, RIF may also increase the expression of certain antioxidant genes, providing another potential mechanism for its health benefits (15). A study conducted by Madkour et al. (30) provides the first evidence of a link between RIF and fat mass and obesity-associated (*FTO*) gene expression in overweight/obese individuals. The authors found an association between reduced *FTO* expression and favorable effects such as suppression of pro-inflammatory markers and improved lipid profile. *FTO* is broadly distributed in many organs, and its expression may be influenced by dietary conditions. The study also found a reduction in pro-inflammatory cytokines such as interleukin (IL)-6, tumor necrosis factor- α (TNF-α), and an increase in IL-10, an anti-inflammatory cytokine, during RIF. These findings are consistent with previous research showing significant reductions in pro-inflammatory cytokines and improvements in cardiometabolic risk factors during RIF (12, 34). The study by Madkour et al. (30) found no correlation between *FTO* expression and high-energy intake, waist circumference, or obesity, suggesting that RIF’s beneficial effects occur independently of dietary and anthropometric factors. The study also found that RIF upregulates several key regulatory proteins involved in tumor suppression, DNA repair, insulin signaling, glucose and lipid metabolism, circadian clock regulation, immune system, and cognitive function. Similarly, another study by Madkour et al. (35) investigated the impact of RIF on the genetic expression of metabolic and cellular regulator genes (*SIRT1* and *SIRT3*) and antioxidant defense enzyme system genes (*TFAM, SOD2,* and *Nrf2*). The research revealed that the expression of SIRT1 shows a minor reduction at the end of RIF, while SIRT3 shows a significant reduction, which could be due to the lack of significant changes in total energy and fat intake during Ramadan. The study further revealed that the expression of antioxidant defense genes (*SOD2, TFAM*, and *Nrf2*) increases, suggesting their role in counteracting the increased oxidative stress during fasting. The research also notes that the significant reduction of IGF-1 reflects the significant activation of antioxidative stress genes, providing a positive transient protective impact against oxidative stress and subsequent pathological conditions.

### Association between PCOS, gut microbiota, and bile acid metabolism

Studies on the gut microbiota may be useful in interpreting the impact of IF, which may be mediated by alterations brought about by IF on the gut microbiota, and in explaining the plausible effect of IF on PCOS. Recent research by Dong and Rees (36) suggests a link between the gut microbiome and the development of PCOS, wherein they reported that women with PCOS exhibit higher levels of *Bacteroides vulgatus* (*B. vulgatus*) and lower levels of certain bile acids in their intestines. Experiments with mice showed that introducing these gut bacteria from PCOS patients or pure *B. vulgatus* led to insulin resistance, altered bile acid metabolism, and disrupted ovarian function. However, administration of IL-22 or glycodeoxycholic acid improved these symptoms, suggesting potential therapeutic strategies. Furthermore, genome-wide association studies have identified various susceptibility loci for PCOS, particularly in metabolic and neuroendocrine pathways. These include loci near genes such as the insulin receptor, follicle-stimulating hormone receptor, and others (36). Additionally, Cienfuegos et al. (32), in their study reported that IF has been found to positively affect the gut microflora’s composition and diversity, and reduce gut permeability, thereby diminishing obesity-linked postprandial endotoxemia and systemic inflammation.

The strength of our review is that is the first comprehensive systematic review that tackles the effect of different forms of IF and CR regimens on the different aspects related to PCOS, including anthropometrics and biochemical parameters including enzymes, glycemic control, lipid profile, hormonal parameters, oxidative stress, and inflammatory markers. However, the study also had some limitations that should be considered when interpreting the findings. Limited research and scarcity of works render the generalizability of the results unattainable. Additionally, the inclusion of an observational study as part of the reviewed articles is another weakness of the study as causality cannot be inferred in such a study design.

## Conclusion

The following review thus presents IF and CR as a subject of much interest and promise in modulating different physiological responses in individuals with PCOS. A scarce of studies have examined the effect of IF and CR on markers of PCOS. Even so, such limited findings demonstrate the complicated and multifaceted nature of the IF regimen and its impact on body composition, metabolic, hormonal, and other biochemical markers related to PCOS. This diversity in outcomes, however, highlights the complex interaction between IF and responses at an individual level. Therefore, the current findings must be interpreted with caution due to these inconsistencies and different outcomes in various studies, alongside the very limited number. Like many developing fields of study, the research on IF highlights the need for more rigorous testing presented in terms of more well-controlled clinical trials. In the future, such efforts should focus on developing comprehensive and well-standardized research protocols; multifaceted populations of women diagnosed with PCOS must be selected for assessment. Longer-term assessments are needed, with more mechanisms revealed. Intermittent fasting may be a modulatory tool for many health parameters, but we must understand this more holistically and nuancedly.

## Data availability statement

The data that support the findings of this study are available from the corresponding author, (MF), upon reasonable request.

## Author contributions

AK: Data curation, Investigation, Software, Writing – original draft, Writing – review & editing. DA: Data curation, Formal analysis, Investigation, Visualization, Writing – original draft, Writing – review & editing. MF: Conceptualization, Data curation, Investigation, Methodology, Project administration, Supervision, Visualization, Writing – original draft, Writing – review & editing.
